# The role of Self Determination Theory and positive classroom experiences in students' mathematics outcomes

**DOI:** 10.3389/fpsyg.2026.1782314

**Published:** 2026-04-10

**Authors:** Isabella M. Santiago, Marley R. Fox, Nicole R. Scalise

**Affiliations:** Department of Human Development, Washington State University, Pullman, WA, United States

**Keywords:** math ability, math anxiety, mathematics education, secondary school, Self-Determination Theory, undergraduate

## Abstract

Many secondary school students hold negative attitudes toward math. This can impact later achievement, attitudes, and decisions about math, including whether to pursue a STEM undergraduate degree or career. Self-Determination Theory (SDT) suggests that students who experience the satisfaction of the basic psychological needs of autonomy, competence, and relatedness are more likely to maintain positive attitudes toward learning, have higher achievement, and demonstrate better overall wellbeing. There is also evidence that emotionally relevant experiences can significantly impact later outcomes, especially in academic contexts. The present study examines the relationship between undergraduate students' previous experiences in secondary school math classes and their current math anxiety, ability, and choice of major. Students whose descriptions of prior math experiences contained more instances of autonomy, competence, and relatedness rated their experiences more positively and reported a higher level of classroom autonomy support. Moreover, students with more positive memories of math from secondary school tended to have lower math anxiety as undergraduate students, with students enrolled in STEM majors reporting significantly more positive secondary school math experiences relative to students enrolled in non-STEM majors. The results provide evidence of an association between positive, supportive secondary school math experiences and undergraduate STEM outcomes.

## 1 Introduction

Many students hold negative attitudes about math or experience math anxiety (Dowker et al., 2016). A recent study of U.S. adults found that on average, adults experience between “some” and “moderate” math anxiety (Hart and Ganley, 2019), while an international study of 15-year-olds found that 59% often worry that mathematics classes will be difficult for them (OECD, 2013). Holding negative attitudes toward math in general, and having math anxiety in particular, predicts lower math achievement and lower rates of enrollment in science, technology, engineering, and mathematics (STEM) courses in college (Daker et al., 2021; Ramirez et al., 2018). Given the prevalence of mathematics anxiety and its relationship with mathematics achievement, it is important to understand the development of math attitudes and abilities over time. Prior mathematical experiences during childhood and adolescence set the stage for later attitudes toward math in emerging adulthood (e.g., John et al., 2020, 2022; Scalise et al., 2025; Towers et al., 2017). In particular, supportive mathematics experiences during adolescence may serve as a “turning point” in the development of math attitudes, informing students' math perspectives as undergraduate students (John et al., 2020). The goal of the present study is to examine the association between prior experiences in secondary mathematics classrooms and undergraduate students' current math attitudes and ability.

Prior research has demonstrated that students who feel supported in academic contexts achieve higher grades and maintain more positive attitudes about learning (Rice et al., 2013). Self-Determination Theory (SDT) suggests that people whose basic psychological needs of autonomy, competence and relatedness are met will have better overall wellbeing relative to people with one or more unmet needs (Deci and Ryan, 1985; Niemiec and Ryan, 2009). Applied to educational contexts, SDT suggests that students who feel that they have a say in their learning (autonomy), succeed at challenging activities (competence), and feel connected, valued, and respected by others (relatedness) will have higher academic achievement and attitudes (Niemiec and Ryan, 2009; Ryan and Deci, 2020). Indeed, a study of secondary school students found that the adolescents with the most needs satisfied demonstrated the highest achievement and most positive affect, as well as the least negative affect and academic stress compared to students with the most dissatisfied needs profile (Earl et al., 2019).

In mathematics education contexts, the three basic psychological needs are key to supporting students' mathematics motivation and achievement (Schukajlow et al., 2017) and decreasing math anxiety (Johnston-Wilder et al., 2021). A study by Kosko (2015) found that secondary school students who report experiencing more autonomy, competence, and relatedness are more engaged in and contribute more frequently to class relative to students with unmet needs. Some prior research has also demonstrated unique contributions of SDT factors to students' mathematics success: Adolescents' perceptions of autonomy within their mathematics classroom significantly predict their broader motivational beliefs and end-of-year math achievement (León et al., 2015), and experiences of competence in mathematics predicts adolescents' interest in math (Schukajlow and Krug, 2014) as well as undergraduate students' mathematics achievement (Wang et al., 2022). Additional research has shown evidence that secondary and undergraduate students' perception of autonomy support, competence, and relatedness predicts their persistence in undergraduate STEM (e.g., initial selection or continued enrollment in a STEM major; Hilts et al., 2018; Simon et al., 2015). Such findings underscore the importance of SDT in students' mathematics outcomes; however, most prior research focuses on short-term associations within a single course or academic term, leaving the possibility of longer-term associations as an open question. Moreover, most prior research measures students' perceptions of the three basic psychological needs using self-report questionnaires (e.g., Learning Climate Questionnaire (LCQ); Bowen and Kilmann, 1975; Black and Deci, 2000; Núñez et al., 2012; Williams and Deci, 1996). Less is known about whether experiences of autonomy, competence, and relatedness are a salient part of students' memories of prior math experiences.

A key tenet of development is that an individual's prior experiences inform their later beliefs, attitudes, and behaviors. Students' experiences with mathematics in secondary school may lay the foundation for their math attitudes and outcomes as undergraduate students. Memories or narratives of math from prior experiences, and their emotional valence, predict current math attitudes. John et al. (2020) found that individuals who reported consistently positive narratives about math tended to be less math anxious and have higher motivation than their counterparts who reported consistently negative math narratives, who were more likely to avoid math altogether. Additionally, similar experiences of math “low points” or challenges may be appraised positively or negatively by different people, depending on the individual's math self-concept (John et al., 2022). This suggests that broader classroom context, including the level of autonomy, competence, and relatedness that a student perceives, may affect the appraisal of math narratives and influence their overall emotional valence. Creating a positive environment around challenging mathematics situations may mitigate a negative appraisal, which consequently may affect a student's attitudes toward mathematics in the future. Understanding the associations between prior math experiences and later math outcomes is critical to study for the purpose of intervention – if undergraduate students' math attitudes and skills are informed by the level of psychological support they received in secondary school, it prompts the need for additional interventions targeting adolescents. Students' past experiences in classrooms may have an impact on not only their future attitudes and achievement, but also their future academic decisions such as course and major enrollment (Chen et al., 2020; Hunt and Maloney, 2022).

The present study examines the relations between undergraduate students' experiences of autonomy, competence, and relatedness in their secondary school math classes, their perceived autonomy support, and their current math anxiety and math ability. We further examine the relation between characteristics of students' secondary school math experiences and their choice of college major (STEM vs. non-STEM). Building from SDT (Deci and Ryan, 1985), we hypothesized that students whose past math experiences included more instances of autonomy, competence, and relatedness would rate their experience more positively and would report a higher level of overall autonomy support relative to students with fewer experiences of autonomy, competence, and relatedness. Further, we hypothesized participants who described more SDT constructs in their memories would demonstrate higher current math ability, lower current math anxiety, and be more likely to be enrolled in a STEM major relative to participants who described fewer SDT constructs. Finally, in keeping with research on the emotional valence of prior math experiences (John et al., 2020, 2022; Scalise et al., 2025), we hypothesized that students' ratings of their emotions during their secondary school math experiences would be significantly associated with their current math outcomes, such that students who described more positive experiences would also, on average, have higher math ability, lower math anxiety, and be more likely to enroll in a STEM major than participants who described a more negative prior math experience.

## 2 Method

### 2.1 Participants

Participants were recruited from a large public northwestern university in the United States from the Department of Human Development research subject pool in exchange for course credit or from introductory STEM classes (Calculus I and Introduction to Electrical Engineering and Computer Science) in exchange for entry into a raffle. otal of 169 eligible participants initially enrolled in the study, however, data from 11 participants was excluded from the analytic sample due to: duplicate entries (i.e., reported the same name on the electronic consent form, *n* = 7) and missing or non-sensical responses on the focal measure describing their memory of a high school math experience (*n* = 4). Any additional missingness on specific measures was excluded in pairwise analyses and retained for analyses where participants had complete responses. 100 and 58 undergraduate students were included in the analytic sample (*M*_*age*_ = 20.4 years, *SD* = 4.4 years, range 18–57 years; 65% female, 29% male, 6% other; 14.6% Hispanic/Latinx, 85.4% non-Hispanic/Latinx; 70.1% White, 8.4% Asian, 7.8% African American or Black, 2.6% Native Hawaiian or Pacific Islander, 0.6% American Indian or Alaskan Native, 7.1% multiracial, 3.2% other; 38.6% first-year, 31.0% sophomore, 13.9% junior, 15.2% senior, 1.3% other). Seventeen percent of participants (*n* = 27) were majoring in a STEM field (Washington State University, n.d.)^1^.

### 2.2 Procedure

Participants completed all measures on a Qualtrics survey. Approximately one-third of participants (*n* = 46) completed the survey in-person in an on-campus lab setting with a trained research assistant. The remaining participants (*n* = 112) completed the survey independently online from a location of their choice. First, participants were asked to sign an informed consent form and asked a series of demographic questions. Next, they were asked to describe their memories of secondary school math, their general perception of autonomy support in the classroom, their mathematics anxiety, and to complete a brief mathematics assessment. All measures were presented in the same order across participants.

### 2.3 Measures

#### 2.3.1 Secondary school math memory

Participants were asked to spend 3 min describing their most vivid or significant memory of an experience in a secondary school math class in a free-response textbox. The time limit was suggested but not enforced (*M* = 2.55 min response time, range = 0.36 −7.65 min). They were asked to include what they did, where they were, who was there, and any thoughts and feelings that they experienced. Responses were coded for instances of autonomy, competence, and relatedness based on their responses with reference to SDT definitions of the constructs (Ryan and Deci, 2000; Niemiec and Ryan, 2009). These codes were not mutually exclusive, and multiple instances of each construct could be coded for a single response. Definitions and examples of autonomy, competence, and relatedness are included in Table 1. All responses were double-coded by the first and second authors, with discrepancies resolved through discussion with the third author. After describing their memory, participants were asked several multiple-choice questions, including rating their emotions during the experience (i.e., very negative, negative, somewhat negative, neutral, somewhat positive, positive, and very positive); what year of secondary school they were in (i.e., freshman, sophomore, junior, senior); what math class they were in during the memory (i.e., pre-algebra, algebra 1, geometry, algebra 2, statistics, trigonometry, pre-calculus, calculus); and whether or not they were with anyone else during the experience (i.e., they were alone; with a teacher; with classmates).

**Table 1:** Definitions and examples of autonomy, competence, and relatedness.

Code	Definition	Example
Autonomy	Demonstrating agency; understanding teacher rationale	“…We had control over…the project…” “…helped us understand how math could be used in real-world experiences.”
Competence (perceived)	Confidence in abilities; positive comparison of abilities in terms of self	“…I felt confident in my answers…” “…a lot of people had trouble with the content and I had relative ease.”
Competence (objective)	Showing evidence of achievement; perception of adequate resources	“…I was getting 100 on most of my tests.” “I remember he would often do this [referencing effective teaching strategy] …make the math easier to understand.”
Relatedness	Feeling supported; positive interactions; feeling respected	“…she made students feel welcomed and loved.” “…I had a lot of friends in my class.”

#### 2.3.2 Perceived autonomy support

Students' perceived level of overall autonomy support in their classroom environments was measured using the 15-item Learning Climate Questionnaire (LCQ; Williams and Deci, 1996). Participants were asked to rate how true situations were to them with respect to their secondary school math experience on a 7-point Likert scale ranging from strongly disagree to strongly agree. Items included “I felt that my teacher accepted me” and “My teachers answered my questions fully and carefully.” Scores were calculated by averaging the individual item scores with a reliability of α = 0.96.

#### 2.3.3 Math anxiety

The 24-item Mathematics Anxiety Rating Scale—Revised (MARS-R; Plake and Parker, 1982), was used to measure participants' mathematics anxiety Participants were asked to rate how anxious certain situations would make them feel, such as “starting a new chapter in a math book”, on a 5-point Likert-type scale ranging from not at all anxious to extremely anxious. Scores were calculated by averaging the individual item scores with overall reliability α = 0.96.

#### 2.3.4 Math ability

Participants' math ability was measured using a 10-item multiple choice math assessment derived from preliminary SAT (PSAT) practice exams from the College Board (The College Board, 2015; Appendix A). Items included concepts from algebra, problem-solving and data analysis, and geometry. Scores were calculated as the total number of items participants answered correctly. Prior research has demonstrated acceptable reliability of PSAT items (α = 0.87; The College board, 2006).

### 2.4 Analytic strategy

We completed several analyses to address our proposed research questions. First, we conducted preliminary analyses using two-tailed *t*-tests to assess the comparability of participants who completed the study in-person vs. independently online. Next, we descriptively summarized characteristics of participants' secondary school math memories and current math ability, math anxiety, and choice of STEM majors. Subsequently, we conducted both exploratory and planned analyses. We conducted two-tailed *t*-tests to determine whether participants' emotions ratings of their prior math experiences varied by participant gender, whether participants were alone or working with someone else, and their grade level in secondary school at the time of the memory. We did not have *a priori* hypotheses about the directionality of these tests. In our planned analyses, we tested explicit, *a priori* hypotheses with a specified direction using one-tailed *t*-tests and correlations. Finally, building from the pattern of associations we observed in our planned analyses, we tested an exploratory structural equation model (SEM) of participants' SDT-related experiences in secondary school math predicting their emotion ratings, which predicted post-secondary math-related outcomes. All preliminary, descriptive, and planned analyses were conducted in *R* (R Core Team, 2024). The exploratory SEM model was conducted in *Mplus* Version 8.6 using maximum likelihood estimation (Muthén and Muthén, 2017).

## 3 Results

### 3.1 Preliminary comparison of in-person vs. online participants

Preliminary *t*-test analyses confirmed that there were no statistically significant differences between participants who completed the survey in-person vs. independently online on their reports of total instances of SDT, perceived autonomy support, math anxiety, or math ability (all *p*s > 0.15).

### 3.2 Characteristics of secondary school math memories

Participants' secondary school math memories varied considerably. Most participants reported a memory from later years in their secondary school experience, with 39% of participants (*n* = 61) recalling an experience in their senior year and one quarter from their junior year (25%, *n* = 40). Fewer participants reported memories from their freshman and sophomore years of secondary school (15%, *n* = 23 and 22%, *n* = 34, respectively). There was also variability in the type of math class participants were taking while the memory occurred: 10% of participants were enrolled in pre-algebra or Algebra 1 (*n* = 16), 15% were taking Geometry (*n* = 23), 22% were taking Algebra 2 (*n* = 34), 25% were taking Trigonometry/Pre-calculus (*n* = 39), 19% were taking Calculus (*n* = 30), 7% were taking Statistics (*n* = 11), and 3% were taking another course (e.g., introduction to college math; *n* = 5).

Most participants described being with other people during their memories of math: 63% reported interacting with their teacher (*n* = 99); 68% reported being around classmates (*n* = 107); and 8% reported being alone during the experience (*n* = 13). When participants were asked to rate their emotions during their experience, responses ranged from 1 (extremely negative) to 7 (extremely positive), with the average rating being between neutral and slightly positive (*M* = 4.45, *SD* = 1.9). There was a statistically significant difference in emotion ratings between female participants and male participants, such that male participants reported higher average emotion ratings relative to female participants (*M*_*female*_ = 4.17, *M*_*male*_ = 5.02, *t* (102.76) = 2.80, *p* < 0.01). Similarly, participants who described being with someone else during their experience rated the experience significantly more positively than participants who described being alone (*M* = 4.55 and *M* = 3.10, respectively; *t* (13.27) = 3.77, *p* < 0.01). However, there was not a significant relation between emotion ratings and grade level during which the memory took place (*r* (147) = 0.09, *p* = 0.25).

### 3.3 Autonomy, competence, and relatedness in math memories

Table 2 describes the frequency of references to autonomy, competence, and relatedness in participants' memories of secondary school math experiences. Instances of each type of construct were somewhat rare: Approximately half of participants (*n* = 77) had one or no references to autonomy, competence, or relatedness, while the remaining participants referred to between two and six instances. As hypothesized, participants whose free response math memory contained more total instances of autonomy, competence, and relatedness tended to rate the memory they described as more positive, *r* (147) = 0.44, one-tailed *p* < 0.001.

**Table 2:** Frequency of references to autonomy, competence, and relatedness.

SDT construct	*M*	*SD*	Min	Max
Autonomy	0.22	0.50	0	2
Competence (perceived)	0.24	0.51	0	3
Competence (objective)	0.45	0.68	0	3
Relatedness	0.79	0.76	0	3
Total	1.70	1.37	0	6

### 3.4 Autonomy support in secondary math classrooms

Participants' ratings of their experience of autonomy support in their math classroom ranged from 1 (strongly disagree) to 7 (strongly agree), with the average rating falling between neutral and slightly agree (*M* = 4.87, *SD* = 1.63). As hypothesized, participants whose memories contained more instances of SDT constructs also rated their overall classroom environment as more autonomy-supportive, *r* (155) = 0.49, one-tailed *p* < 0.001.

### 3.5 Relations between secondary math experiences and undergraduate math outcomes

Participants ranged in thseir reported levels of math anxiety and math ability (Table 3). Participants reported between slight and moderate anxiety about math. On average, participants were 62% correct on the math ability measure. Seventeen percent of participants (*n* = 27) were categorized as STEM majors and the remaining 83% were categorized as non-STEM majors.

**Table 3:** Descriptive statistics of participants' math anxiety and ability.

Outcome	*M*	*SD*	Potential range	Range
Math anxiety	2.51	0.87	1–5	1–5
Math ability	6.2	2.3	0–10	1–10

Contrary to our hypotheses, the number of SDT constructs described was not significantly related to participants' current math ability (*r* (155) = −0.03, one-tailed *p* = 0.37) nor their current math anxiety (*r* (145) = .14, one-tailed *p* = 0.05). Students who described more SDT constructs in their secondary school math memories were not more likely to be enrolled in a STEM major, *M*_*STEM*_ = 1.78, *M*_*Non*−*STEM*_= 1.69, *t* (156) = 0.31, one-tailed *p* = 0.38.

We also examined whether participants' ratings of their emotions during secondary school math experiences was associated with their current math anxiety, ability, and choice of STEM major. Building on prior research on undergraduate students' earliest memories of math and current math abilities, attitudes, and decisions (Scalise et al., 2025), we hypothesized that participants with more positive memories of math in secondary school would report lower math anxiety, higher math ability, and be more likely to major in STEM relative to participants with more negative memories of math. We found evidence of a negative association between participants' ratings of their emotions during their math experience and their current math anxiety, *r* (137) = -.17, one-tailed *p* = 0.027). Participants who were STEM majors also had significantly more positive emotion ratings of their secondary school math experiences relative to non-STEM majors, *M*_*STEM*_ = 5.52, *M*_*Non*−*STEM*_ = 4.21, *t* (147) = 3.34, one-tailed *p* = 0.0005. However, there was no association between participants' ratings of their emotions during their experience and their current math ability (*r* (146) = .004, one-tailed *p* = 0.48).

**Figure 1:**
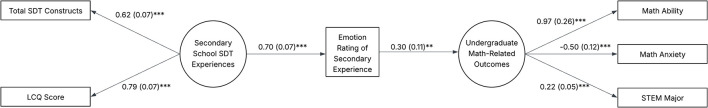
Structural equation model of secondary experiences and undergraduate math outcomes. All estimates are standardized, with standard errors provided within parentheses. ^**^p < 0.01; ^***^p < 0.001.

Building from the results of the planned analyses, we conducted an exploratory SEM model to estimate multiple effects simultaneously. Our exploratory model included two latent factors—a factor representing SDT experiences in secondary school from total SDT constructs and LCQ scores, and a factor for undergraduate math-related outcomes from math ability scores, math anxiety scores, and enrollment in a STEM major as indicators. Our model included a direct path from the SDT latent factor to a measured variable for participants' emotion rating of their secondary math experience, as well as a direct path from participants' emotion rating to the undergraduate math outcomes factor (Figure 1). The chi-square for the model was significant significant (χ^2^(8) = 18.11, *p* = 0.02) and the Comparative Fit Index (CFI) was 0.932, approaching the benchmark value of 0.95 (Hu and Bentler, 1999) suggesting the model explains a large amount of variance in the data relative to a null model. The SRMR was 0.056, less than the benchmark value of 0.08 (Hu and Bentler, 1999), suggesting there is little variance left unexplained in the data after accounting for the associations in the model. These results suggest overall adequate data-model fit. As shown in Figure 1, the direct effects between SDT experiences and participants' emotion ratings and emotion ratings and undergraduate math outcomes were statistically significant and moderate in magnitude.

## 4 Discussion

Emerging adults attending university are faced with many decisions related to course enrollment, major, and career pathways. Students' mathematical abilities and attitudes serve as a critical gateway to their access to higher-paying STEM professions (Watt and Goos, 2017); however, students enter college with these abilities and attitudes pre-formed. The goal of the present study was to examine the role of previous math experiences in secondary school on undergraduate students' current math abilities and attitudes. We found support for our hypothesis that the three basic psychological needs described in SDT are important to creating a positive secondary school math experience for students: The more instances of SDT constructs described in each memory, the more likely participants were to rate it positively. Similarly, the number of SDT constructs present in students' memories was predictive of their overall perceived autonomy support. In contrast, we found that there was no bivariate relationship between the number of SDT constructs in students' secondary school math memories and their current math anxiety or ability. In addition, the number of SDT constructs described by students did not predict whether participants were enrolled in a STEM major. However, we found that students with more positive secondary school math experiences had lower math anxiety and were more likely to be enrolled in a STEM major relative to students with less positive math experiences. When combined into an exploratory SEM model, we found support for direct pathways between students' SDT-related experiences in secondary math to positive emotion ratings, and in turn, positive emotion ratings of secondary math experiences to undergraduate math outcomes.

Our findings provide support for the importance of secondary school students' experiences of autonomy, competence, and relatedness in math classes. When students perceived these types of support in their secondary school math class, they appraised their experience more positively. Indeed, studies of SDT interventions have found that when teachers are trained to provide more autonomy supportive instruction, their secondary school students demonstrate more classroom engagement, skill development, and academic achievement relative to control groups (Cheon et al., 2012, 2020). Meta-analyses of SDT-aligned interventions in classroom contexts have shown consistent, positive effects on students' outcomes (Lazowski and Hulleman, 2016; Reeve and Cheon, 2021).

Most prior studies of students' experiences of autonomy, competence, and relatedness rely on self-report questionnaires (e.g., Cheon et al., 2018; Wang et al., 2022). The present study applied a novel coding scheme to students' open-ended descriptions of their mathematics experiences and found evidence that some students do recall instances of autonomy, competence, and relatedness unprompted. Moreover, we found evidence of construct validity for our SDT coding scheme, such that students' experiences of autonomy, competence, and relatedness significantly correlated with their perceived autonomy support assessed by a standard SDT questionnaire. Although our coding scheme was applied to a singular memory of a secondary school math experience, the LCQ is intended to measure overall perceived autonomy support within a class. This suggests that asking students to recall a specific but salient memory may be generally associated with their year-long perceptions of their experiences within their math class. Indeed, theories of memory development suggest that similar experiences that raise similar emotional reactions over time become significant self-defining memories (John et al., 2022; Singer, 1995). Such prior experiences play a major role in shaping students' attitudes and beliefs, especially in learning contexts (e.g., John et al., 2020; Scalise et al., 2025).

Contrary to our hypotheses, we found that students' experiences of SDT constructs in secondary school math memories were not directly related to their current math attitudes, ability, and choice of STEM major. There are several potential explanations for these findings. First, only half of our sample described any examples of autonomy, competence, and/or relatedness in their description of their prior math experience. It is possible that a more targeted prompt (e.g., “Describe a time in which you felt that you either had a choice or lacked a choice in your learning experience”) would yield more overall reports of SDT constructs and more variability between participants. Second, it is possible that the associations between SDT in prior classroom experiences and undergraduate-level outcomes are simply small in magnitude, requiring a larger sample size to detect accurately (see Limitations). Finally, it is possible that students' prior experiences of SDT are not directly associated with their later ability, anxiety, and course decision-making. Although prior experiences lay the foundation for future skills and attitudes (John et al., 2022; Chen et al., 2020), factors other than SDT constructs may explain the connections between prior and current math experiences. Students' past experiences within the same academic term, year, or undergraduate tenure may have stronger associations with their math outcomes since they are more recent and salient. Moreover, our exploratory SEM model suggests that students' emotion ratings of their secondary math experiences were more directly associated with their undergraduate outcomes than their level of SDT experiences. It is also possible that other environmental factors may be more relevant to undergraduate students' attitudes about math—such as their history of math performance and anxiety (Field et al., 2019; Hunt and Maloney, 2022), their exposure to and knowledge of STEM careers (Blotnicky et al., 2018), their intersecting identities and level of stereotype threat (Beasley and Fischer, 2012; Cundiff et al., 2013), or their general appraisal of their prior math experiences (John et al., 2020; Scalise et al., 2025).

In keeping with prior literature, we found evidence that students' ratings of their emotions during prior math experiences related to their current math outcomes as undergraduates (John et al., 2020; Scalise et al., 2025). Students who rated their secondary school math experiences more positively had lower rates of math anxiety as undergraduates, and students majoring in STEM had more positive average ratings of their emotions during previous math experience relative to students in non-STEM majors. These findings underscore the importance of considering earlier experiences in students' mathematics careers as gateways (or gatekeepers) to their later math and STEM success. Our findings suggest that experiences of autonomy, competence, and relatedness may play some role in students' positive perceptions. According to SDT, students who have more of their basic psychological needs met in a specific class context are more likely to thrive academically and emotionally (Earl et al., 2019). It is possible that the associations predicted by SDT form a mediation when assessed over time, such that experiences of SDT constructs in secondary school lead to stronger math abilities and more positive general attitudes toward math, which mediate the association with later math outcomes in college. Our results further suggest a second factor associated with students' ratings of their emotions during prior math experiences: Level of social interaction. It is possible that more socially interactive secondary school math experiences, which may also yield more experiences of relatedness, are a stronger predictor of more positive math memories. Indeed, social interactivity is a core component of many seminal theories of learning, motivation, and achievement (e.g., Sociocultural Theory; Social Learning Theory; Social Cognitive Theory; Expectancy-Value Theory). Future research should unpack the factors that predict students' more positive appraisals of secondary school math experiences.

### 4.1 Limitations and future directions

There are several limitations to the present study that should be addressed in future research. First, future research should seek to replicate and extend the present findings with larger and more representative samples. Moreover, there may be moderating effects of these associations depending on students' demographic characteristics. For example, it is possible that minoritized students may benefit more from supportive prior experiences, given the barriers many experience in gaining access to STEM fields (Estrada et al., 2016; Kricorian et al., 2020). Second, although one of our goals was to characterize students' recollections of SDT constructs in prior experiences unprompted, future research may benefit from asking more specifically about students' experiences of autonomy, competence, and relatedness in prior math settings. Many participants wrote about a broader, less concise memory when they were asked about the “most vivid or significant experience” in a secondary school math classroom. Relatedly, the present coding scheme was designed to capture positive, supportive educational environments. Future research should further examine participants' responses for negative experiences (e.g., high control or autonomy-thwarting experiences). Third, the present study used a single timepoint, correlational design. Future research should examine evidence for causal associations over time by implementing classroom SDT interventions and assessing students' engagement, attitudes, and overall learning across multiple timepoints. This type of longitudinal experimental design would provide further clarity on the directionality of any observed associations. In doing so, it would reduce concerns of potential confounding order effects from the present design (e.g., rating their emotions during prior math experiences may have affected participants' responses on measures of math anxiety and ability; c.f. Scalise et al., 2025 which showed no order effects of these measures using a counterbalanced design). Finally, future research should examine the representativeness of retrospective narratives of singular learning experiences. Our results suggest some evidence of overlap between students' memories of secondary school math and their general perceptions of autonomy support across the school year. However, a single experience may not be sufficient to capture associations with students' future anxiety, ability, and academic decisions. It is perhaps more likely that broad perceptions of classroom environments over the course of many experiences may be more strongly associated with future outcomes.

## Data Availability

The raw data supporting the conclusions of this article will be made available by the authors, without undue reservation.

## Footnotes

1 The following undergraduate majors are considered STEM fields at Washington State University, based on the definition from the U.S. National Science Foundation (NSF): Agricultural Biotechnology; Animal Sciences; Earth Sciences (Geology); Environmental and Ecosystem Sciences; Field Crop Management; Food Science; Forestry Ecology & Management; Fruit and Vegetable Management; Integrated Plant Sciences; Wildlife Ecology and Conservation Sciences; Winemaking (Viticulture and Enology); Biology; Chemistry; Data Analytics; Human Biology; Mathematics; Physics and Astronomy; Zoology; Architectural Studies; Bioengineering; Chemical Engineering; Civil Engineering; Computer Engineering; Computer Science; Construction Engineering; Construction Management; Electrical Engineering; Landscape Architecture; Materials Science Engineering; Software Engineering; Biochemistry; Genetics and Cell Biology; Microbiology; Neuroscience.

## References

[B1] Beasley M. A. Fischer M. J. (2012). Why they leave: the impact of stereotype threat on the attrition of women and minorities from science, math and engineering majors. Soc. Psychol. Educ. 15, 427–448. doi: 10.1007/s11218-012-9185-3

[B2] Black A. E. Deci E. L. (2000). The effects of instructors' autonomy support and students' autonomous motivation on learning organic chemistry: a self-determination theory perspective. Sci. Educ. 84(6), 740-756. doi: 10.1002/1098-237X(200011)84:6<740::AID-SCE4>3.0.CO;2-3

[B3] Blotnicky K. A. Franz-Odendaal T. French F. Joy P. (2018). A study of the correlation between STEM career knowledge, mathematics self-efficacy, career interests, and career activities on the likelihood of pursuing a STEM career among middle school students. Int. J. STEM Educ. 5:22. doi: 10.1186/s40594-018-0118-3 30631712 PMC6310414

[B4] Bowen D. D. Kilmann R. H. (1975). Developing a comparative measure of the learning climate in professional schools. J. Appl. Psychol. 60, 71–79. doi: 10.1037/h0076363

[B5] Chen C. Kang J. M. Sonnert G. Sadler P. M. (2020). High school calculus and computer science course taking as predictors of success in introductory college computer science. ACM Trans. Comput. Educ. 21, 1–21. doi: 10.1145/3433169

[B6] Cheon S. H. Reeve J. Lee Y. Lee J. (2018). Why autonomy-supportive interventions work: explaining the professional development of teachers' motivating style. Teach. Teach. Educ. 69, 43–51. doi: 10.1016/j.tate.2017.09.022

[B7] Cheon S. H. Reeve J. Moon I. S. (2012). Experimentally based, longitudinally designed, teacher-focused intervention to help physical education teachers be more autonomy supportive toward their students. J. Sport Exerc. Psychol. 34, 365–396. doi: 10.1123/jsep.34.3.365 22691399

[B8] Cheon S. H. Reeve J. Vansteenkiste M. (2020). When teachers learn how to provide classroom structure in an autonomy-supportive way: benefits to teachers and their students. Teach. Teach. Educ. 90:103004. doi: 10.1016/j.tate.2019.103004

[B9] Cundiff J. L. Vescio T. K. Loken E. Lo L. (2013). Do gender–science stereotypes predict science identification and science career aspirations among undergraduate science majors? Soc. Psychol. Educ. 16, 541–554. doi: 10.1007/s11218-013-9232-8

[B10] Daker R. J. Gattas S. U. Sokolowski H. M. Green A. E. Lyons I. M. (2021). First-year students' math anxiety predicts STEM avoidance and underperformance throughout university, independent of math ability. Npi Sci. Learn. 6:17. doi: 10.1038/s41539-021-00095-7 PMC8203776 34127672

[B11] Deci E. L. Ryan R. M. (1985). Intrinsic Motivation and Self-Determination in Human Behavior. New York, NY: Plenum.

[B12] Dowker A. Sarkar A. Looi C. Y. (2016). Mathematics anxiety: what have we learned in 60 years? Front. Psychol. 7:00508. doi: 10.3389/fpsyg.2016.00508 27199789 PMC4842756

[B13] Earl S. R. Taylor I. M. Meijen C. Passfield L. (2019). Young adolescent psychological need profiles: associations with classroom achievement and wellbeing. Psychol. Sch. 56, 1004–1022. doi: 10.1002/pits.22243

[B14] Estrada M. Burnett M. Campbell A. G. Campbell P. B. Denetclaw W. F. Gutiérrez C. G. . (2016). Improving underrepresented minority student persistence in STEM. CBE Life Sci. Educ. 15:es5. doi: 10.1187/cbe.16-01-0038 27543633 PMC5008901

[B15] Field A. P. Evans D. Bloniewski T. Kovas Y. (2019). Predicting maths anxiety from mathematical achievement across the transition from primary to secondary education. R. Soc. Open Sci. 6:191459. doi: 10.1098/rsos.191459 31827871 PMC6894589

[B16] Hart S. A. Ganley C. M. (2019). The nature of math anxiety in adults: prevalence and correlates. J. Numer. Cogn. 5, 122–139. doi: 10.5964/jnc.v5i2.195 33842689 PMC8034611

[B17] Hilts A. Part R. Bernacki M. L. (2018). The roles of social influences on student competence, relatedness, achievement, and retention in STEM. Sci. Educ. 102, 744–770. doi: 10.1002/sce.21449

[B18] Hu L. Bentler P. M. (1999). Cutoff criteria for fit indexes in covariance structure analysis: conventional criteria vs. new alternatives. Struct. Equ. Model. 6, 1–55. doi: 10.1080/10705519909540118

[B19] Hunt T. E. Maloney E. A. (2022). Appraisals of previous math experiences play an important role in math anxiety. Ann. N. Y. Acad. Sci. 1515, 143–154. doi: 10.1111/nyas.14805 35668556 PMC9796677

[B20] John J. E. Nelson P. A. Klenczar B. Robnett R. D. (2020). Memories of math: narrative predictors of math affect, math motivation, and future math plans. Contemp. Educ. Psychol. 60:101838. doi: 10.1016/j.cedpsych.2020.101838

[B21] John J. E. Vierra K. D. Robnett R. D. (2022). I have cried in almost all of my math classes. relations between math self-concept, gender, and narrative appraisals of past low points in math. Contemp. Educ. Psychol. 70:102094. doi: 10.1016/j.cedpsych.2022.102094

[B22] Johnston-Wilder S. Lee C. Mackrell K. (2021). Addressing mathematics anxiety through developing resilience: building on self-determination theory. Creat. Educ. 12, 2019–2115. doi: 10.4236/ce.2021.129161

[B23] Kosko K. W. (2015). Geometry students' self-determination and their engagement in mathematics whole class discussion. Investig. Math. Learn. 8, 17–36. doi: 10.1080/24727466.2015.11790349

[B24] Kricorian K. Seu M. Lopez D. Ureta E. Equils O. (2020). Factors influencing participation of underrepresented students in STEM fields: matched mentors and mindsets. Int. J. STEM Educ. 7:16. doi: 10.1186/s40594-020-00219-2

[B25] Lazowski R. A. Hulleman C. S. (2016). Motivation interventions in education. Rev. Educ. Res. 86, 602–640. doi: 10.3102/0034654315617832

[B26] León J. Núñez J. L. Liew J. (2015). Self-determination and STEM education: effects of autonomy, motivation, and self-regulated learning on high school math achievement. Learn. Individ. Differ. 43, 156–163. doi: 10.1016/j.lindif.2015.08.017

[B27] Muthén L. K. Muthén B. O. (2017). Mplus User's Guide, Edn.*8*. Los Angeles, CA: Muthén & Muthén.

[B28] Niemiec C. P. Ryan R. M. (2009). Autonomy, competence, and relatedness in the classroom: applying self-determination theory to educational practice. Theory Res. Educ. 7, 133–144. doi: 10.1177/1477878509104318

[B29] Núñez J. L. León J. Grijalvo F. Albo J. M. (2012). Measuring autonomy support in university students: the Spanish version of the learning climate questionnaire. Span. J. Psychol. 15, 1466–1472. doi: 10.5209/rev_SJOP.2012.v15.n3.39430 23156948

[B30] OECD (2013). Mathematics Self-Beliefs and Participation in Mathematics-Related Activities, in PISA 2012 Results: Ready to Learn (Volume III): Students' Engagement, Drive and Self-Beliefs. Paris: OECDPublishing.

[B31] Plake B. S. Parker C. S. (1982). The development and validation of a revised version of the mathematics anxiety rating scale. Educ. Psychol. Meas. 42, 551–557. doi: 10.1177/001316448204200218

[B32] R Core Team, (2024). R: A Language and Environment for Statistical Computing. R Foundation for Statistical Computing, Vienna, Austria. Available online at: https://www.R-project.org. (Accessed March 1, 2024).

[B33] Ramirez G. Shaw S. T. Maloney E. A. (2018). Math anxiety: past research, promising interventions, and a new interpretation framework. Educ. Psychol. 53, 145–164. doi: 10.1080/00461520.2018.1447384

[B34] Reeve J. Cheon S. H. (2021). Autonomy-supportive teaching: its malleability, benefits, and potential to improve educational practice. Educ. Psychol. 56, 54–77. doi: 10.1080/00461520.2020.1862657

[B35] Rice L. Barth J. M. Guadagno R. E. Smith G. P. A. McCallum D. M. (2013). The role of social support in students' perceived abilities and attitudes toward math and science. J. Youth Adolesc. 42, 1028–1040. doi: 10.1007/s10964-012-9801-8 22890901

[B36] Ryan R. M. Deci E. L. (2000). Intrinsic and extrinsic motivations: classic definitions and new directions. Contemp. Educ. Psychol. 25, 54–67. doi: 10.1006/ceps.1999.1020 10620381

[B37] Ryan R. M. Deci E. L. (2020). Intrinsic and extrinsic motivation from a self-determination theory perspective: definitions, theory, practices, and future directions. Contemp. Educ. Psychol. 61:101860. doi: 10.1016/j.cedpsych.2020.101860

[B38] Scalise N. R. Santiago I. Canning E. (2025). When we were young: Memories of early mathematics experiences. J. Numer. Cogn. 11:e14771. doi: 10.5964/jnc.14771

[B39] Schukajlow S. Krug A. (2014). Are Interest and Enjoyment Important for Students' Performance? International Group for the Psychology of Mathematics Education. Available online at: https://files.eric.ed.gov/fulltext/ED600033.pdf

[B40] Schukajlow S. Rakoczy K. Pekrun R. (2017). Emotions and motivation in mathematics education: theoretical considerations and empirical contributions. ZDM 49, 307–322. doi: 10.1007/s11858-017-0864-6 PMC9845103 36684477

[B41] Simon R. A. Aulls M. W. Dedic H. Hubbard K. Hall N. (2015). Exploring student persistence in STEM programs: a motivational model. Can. J. Educ. 38, 1–27. doi: 10.2307/canajeducrevucan.38.2.01

[B42] Singer J. A. (1995). Seeing one's self: locating narrative memory in a framework of personality. J. Pers. 63, 429–457. doi: 10.1111/j.1467-6494.1995.tb00502.x 7562361

[B43] The College board, (2006). Research Report No. 2006-6. Relationships between PSAT/NMSQT scores and academic achievement in high school. Available online at: https://files.eric.ed.gov/fulltext/ED562814.pdf (Accessed December 28, 2006).

[B44] The College Board, (2015). 2015 Practice Test #1: PSAT/NMSQT. Available online at: https://satsuite.collegeboard.org/psat-nmsqt/preparing/practice-tests. (Accessed June 1, 2023).

[B45] Towers J. Hall J. Rapke T. Martin L. C. Andrews H. (2017). Autobiographical accounts of students' experiences learning mathematics: a review. Can. J. Sci. Math. Technol. Educ. 17, 152–164. doi: 10.1080/14926156.2016.1241453

[B46] Wang C. Cho H. J. Bonem E. M. Levesque-Bristol C. Wiles B. Moss J. D. . (2022). Competence and autonomous motivation as motivational predictors of college students' mathematics achievement: from the perspective of self-determination theory. Int. J. STEM Educ. 9, 1–14. doi: 10.1186/s40594-022-00359-7

[B47] Washington State University, (n.d.). STEM Colleges & Majors at WSU. WSU Louis Stokes Alliance for Minority Participation (LSAMP). Available online at: https://lsamp.wsu.edu/stem-colleges-and-majors (Accessed June 1, 2023).

[B48] Watt H. M. G. Goos M. (2017). Theoretical foundations of engagement in mathematics. Math. Educ. Res. J. 29, 133–142. doi: 10.1007/s13394-017-0206-6

[B49] Williams G. C. Deci E. L. (1996). Internalization of biopsychosocial values by medical students: a test of self-determination theory. J. Pers. Soc. Psychol. 70, 767–779. doi: 10.1037/0022-3514.70.4.767 8636897

