# Supplementary material for: The role of Self Determination Theory and positive classroom experiences in students' mathematics outcomes
Source: Front Psychol. 2026 Apr 10;17:1782314. doi: 10.3389/fpsyg.2026.1782314 (PMC13106378; doi:10.3389/fpsyg.2026.1782314)
Supplement: Supplementary file 1 [file Supplementary_file_1.docx]

# Appendix A: Math Ability Measure

For the following section, you will be prompted to complete a 10 question math test. You may take as much time as you need to complete these questions. However, please refrain from using a calculator since all questions can be solved without one. Make your best guess if you are unsure of the correct answer.

1. If $\frac{x-1}{3}=k$ and $k=3$, what is the value of $x$?
   1. 2
   2. 4
   3. 9
   4. 10
2. For $i= \surd(-1)$, what is the sum $\left( 7+3i \right)+(-8+9i)$?
   1. $-1+12i$
   2. $-1-6i$
   3. $15+12i$
   4. $15-6i$
3. On Saturday afternoon, Armand sent *m* text messages each hour for 5 hours, and Tyrone sent *p* text messages each hour for 4 hours. Which of the following represents the total number of messages sent by Armand and Tyrone on Saturday afternoon?
   1. 9*mp*
   2. 20*mp*
   3. 5*m* + 4*p*
   4. 4*m* + 5*p*
4. Kathy is a repair technician for a phone company. Each week, she receives a batch of phones that need repairs. The number of phones that she has left to fix at the end of the day can be estimated with the equation $P=108-23d$, where $P$ is the number of phones left and $d$ is the number of days she has worked that week. What is the meaning of the value 108 in this equation?
   1. Kathy will need to complete the repairs within 108 days.
   2. Kathy starts each week with 108 phones to fix.
   3. Kathy repairs phones at a rate of 108 per hour.
   4. Kathy repairs phones at a rate of 108 per day.
5. A line on the *xy-*plane passes through the origin and has a slope of $\frac{1}{7}$. Which of the following points lies on the line?
   1. (0, 7)
   2. (1, 7)
   3. (7, 7)
   4. (14, 2)
6. A babysitter earns $8 an hour for babysitting 2 children and an additional $3 tip when both children are put to bed on time. If the babysitter fets the children to bed on time, what expression could be used to determine how much the babysitter earned?
   1. 8*x* + 3, where *x* is the number of hours
   2. 3*x* + 8, where *x* is the number of hours
   3. *x*(8 + 2) + 3, where *x* is the number of children
   4. 3*x* + (8 + 2), where *x* is the number of children
7. $3\left( x+y \right)=y$

If $(x,y)$ is a solution to the equation above and $y\neq0$, what is the ratio $\frac{x}{y}$?

- 1. $-\frac{4}{3}$
  2. $-\frac{2}{3}$
  3. $\frac{1}{3}$
  4. $\frac{2}{3}$

1. In a game, a player can solve easy or hard puzzles. A player earns 30 points for solving an easy puzzle and 60 points for solving a hard puzzle. Tina solved a total of 50 puzzles playing this game. She earned 1950 points total. How many hard puzzles did Tina solve?
   1. 10
   2. 15
   3. 25
   4. 35


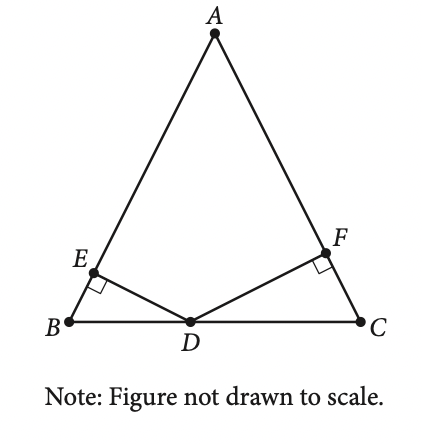


1. Triangle *ABC* above is isosceles with *AB* = *AC* and *BC* = 48. The ratio of *DE* to *DF* is 5 : 7. What is the length of *DC*?
   1. 12
   2. 20
   3. 24
   4. 28
2. Which of the following is equivalent to $(s-t)(\frac{S}{t})$?
   1. $\frac{S}{t}-s$
   2. $\frac{S}{t}-st$
   3. $\frac{s^{2}}{t}-s$
   4. $\frac{s^{2}}{t}-\frac{s}{t^{2}}$
